# Increased richness and diversity of the vaginal microbiota and spontaneous preterm birth

**DOI:** 10.1186/s40168-018-0502-8

**Published:** 2018-06-28

**Authors:** Aline C. Freitas, Alan Bocking, Janet E. Hill, Deborah M. Money, the VOGUE Research Group, Deborah Money, Alan Bocking, Sean Hemmingsen, Janet Hill, Gregor Reid, Tim Dumonceaux, Gregory Gloor, Matthew Links, Kieran O’Doherty, Patrick Tang, Julianne van Schalkwyk, Mark Yudin

**Affiliations:** 1 Department of Veterinary Microbiology, University of Saskatchewan, Saskatoon, SK S7N 5B4 Canada; 2 Departments of Obstetrics and Gynaecology and Physiology, University of Toronto, Toronto, ON M5G 1L4 Canada; 3 Lunenfeld-Tanenbaum Research Institute, M5T1X5, Toronto, ON Canada; 4 Department of Obstetrics and Gynaecology, University of British Columbia, Vancouver, BC V6T 1Z4 Canada; 5 Women’s Health Research Institute, BC Women’s Hospital & Health Centre, Vancouver, BC V6H 3N1 Canada

**Keywords:** Microbiome, Vagina, Lactobacillus, CST, Diversity, Richness, Mollicutes, Preterm birth, Pregnancy, Infection

## Abstract

**Background:**

The bacterial community present in the female lower genital tract plays an important role in maternal and neonatal health. Imbalances in this microbiota have been associated with negative reproductive outcomes, such as spontaneous preterm birth (sPTB), but the mechanisms underlying the association between a disturbed microbiota and sPTB remain poorly understood. An intrauterine infection ascending from the vagina is thought to be an important contributor to the onset of preterm labour. Our objective was to characterize the vaginal microbiota of pregnant women who had sPTB (*n* = 46) and compare to those of pregnant women who delivered at term (*n* = 170). Vaginal swabs were collected from women at 11–16 weeks of gestational age. Microbiota profiles were created by PCR amplification and pyrosequencing of the *cpn*60 universal target region.

**Results:**

Profiles clustered into seven community state types: I (*Lactobacillus crispatus* dominated), II (*Lactobacillus gasseri* dominated), III (*Lactobacillus iners* dominated), IVA (*Gardnerella vaginalis* subgroup B or mix of species), IVC (*G. vaginalis* subgroup A dominated), IVD (*G. vaginalis* subgroup C dominated) and V (*Lactobacillus jensenii* dominated). The microbiota of women who experienced preterm birth (< 37 weeks gestation) had higher richness and diversity and higher Mollicutes prevalence when compared to those of women who delivered at term. The two groups did not cluster according to CST, likely because CST assignment is driven in most cases by the dominance of one particular species, overwhelming the contributions of more rare taxa. In conclusion, we did not identify a specific microbial community structure that predicts sPTB, but differences in microbiota richness, diversity and Mollicutes prevalence were observed between groups.

**Conclusions:**

Although a causal relationship remains to be determined, our results confirm previous reports of an association between Mollicutes and sPTB and further suggest that a more diverse microbiome may be important in the pathogenesis of some cases.

**Electronic supplementary material:**

The online version of this article (10.1186/s40168-018-0502-8) contains supplementary material, which is available to authorized users.

## Background

Preterm birth is defined as delivery before 37 completed weeks of gestational age [1] and can be further sub-categorized in extremely preterm (≤ 27^+6^ weeks^+days^), very preterm (28 to 31^+6^) and late preterm (32 to 36^+6^) [2]. Preterm birth comprises 11% of all livebirths worldwide, and its complications are estimated to cause 35% of world’s neonatal deaths, which represents 3.1 million deaths annually [3]. Children who are born prematurely also have higher rates of cardiovascular disorders, respiratory distress syndrome, neurodevelopmental disabilities and learning difficulties compared with children born at term [4].

Preterm birth is a complex multi-factorial condition with several known risk factors, such as low and high maternal ages [5–7], low BMI [8], black ethnicity [9], tobacco use, heavy alcohol intake, illicit drug use [4], close temporal proximity to a previous delivery [10], and multiple gestation [11]. Although studied extensively, some preterm cases remain unexplained for women with no known risk factors. Intrauterine infection with organisms ascending from the vagina has been hypothesized as an important contributor to preterm birth since many organisms isolated from the amniotic fluid/membranes of women who experienced preterm birth are also found in the lower genital tract of pregnant women [12–15]. A large number of studies support this hypothesis based on the strong association between intra-amniotic bacterial infection and preterm birth [12, 14–20].

The microbiological diagnosis of a ‘normal’ or disturbed vaginal microbiota has historically been based on the Nugent score, the current gold standard diagnostic method that relies on Gram stain of vaginal smears [21]. The ‘normal’ vaginal microbiota in non-pregnant reproductive aged women is understood to be dominated by *Lactobacillus* species, while an abnormal microbiota (defined as bacterial vaginosis) is characterized by low abundance of lactobacilli and an overgrowth of anaerobic bacteria, such as *Gardnerella vaginalis, Prevotella* spp., *Bacteroides* spp., *Mobiluncus* spp. and *Mycoplasma hominis* [22]. In low-risk pregnant women, it has been shown that the vaginal microbiota has reduced richness and diversity and increased abundance of lactobacilli compared to those of non-pregnant women [23–27]. An abnormal microbiota has been previously associated with preterm birth [28], but only a few in depth culture-independent studies of the vaginal microbiota of women who had preterm birth have been published, with inconsistent conclusions [29–32].

The objective of this study was to assess whether there are differences in the vaginal microbiota composition, early in gestation, of women who had spontaneous preterm birth (sPTB) and term delivery that could be further investigated as diagnostic indicators of preterm birth risk. Microbiome profiling was based on sequencing of the *cpn*60 universal target, which provides higher resolution than 16S rRNA variable regions [33] and allows the resolution of *Gardnerella vaginalis* subgroups, a hallmark bacteria in the disturbed microbiota [34].

## Methods

### Study population and sampling

This retrospective cohort study analysed the vaginal microbiota of women who experienced spontaneous preterm birth (sPTB) and compared the resulting microbial profiles to those of pregnant women who delivered at term. The bacterial profiles of pregnant Canadian women at low risk of sPTB who had term deliveries (*n* = 170) were previously generated by our research group [24]. The vaginal microbial profiles of Canadian women who had preterm birth originated from samples of this previous study (*n* = 7) [24] and from the Ontario Birth Study (*n* = 39), resulting in 46 samples. The Ontario Birth Study (ontariobirthstudy.com) is an open longitudinal pregnancy cohort at Mount Sinai Hospital, Toronto, Canada. It is a platform for studies of both pregnancy complications as well as Developmental Origins of Health and Disease related research. The PTB rates for the low-risk cohort and OBS cohorts were 4 and 6.2%, respectively. All biospecimens, including maternal vaginal swabs and maternal and infant blood, are collected concurrently with routine clinical specimens to reduce the burden on study participants. Detailed demographic and lifestyle characteristics are obtained from women during pregnancy and postpartum, and clinical information is extracted from the health records. For the purposes of this report, self-administered vaginal swabs were taken at 16 weeks gestation and placed in dry tubes prior to being placed in − 80 °C for storage in the Lunenfeld Tanenbaum Research Institute Biospecimen Storage and Processing Laboratory. Specimens from all cohorts were processed similarly in terms of sample collection, storage, DNA extraction, library preparation and sequencing.

Clinical and behavioural questionnaire data (pregnancy history, family and personal medical history, psychosocial health, demographic factors and other lifestyle and environmental exposures) were transferred to the Research Electronic Data Capture (REDCap) database protected by a secure server [35]. For the PTB group, eligible participants for this study were women who had undergone preterm delivery at greater than 20 weeks but less than 37 weeks gestational age, where onset of labour occurred spontaneously or in association with cervical incompetence or preterm premature rupture of membranes (PPROM). Vaginal swabs collected from pregnant women (both PTB and term groups) at 11–16 weeks of gestational age were used for bacterial genomic analysis.

Total nucleic acid was extracted from swabs using the MagMAX™ Total Nucleic Acid Isolation Kit (Life Technologies, Burlington, ON, Canada) as per manufacturer’s instructions. Kit reagents are aliquoted to eliminate repeated accessing of open reagents, and samples are processed in small batches using filter tips to prevent cross-contamination. Pipettes and other lab surfaces are regularly treated with DNA surface decontaminant (DNA Away, Thermo Fisher Scientific, Waltham, MA). Samples from both cohorts were processed in exactly the same way in terms of swab type, storage temperature (no stabilizer was used), DNA extraction, library preparation and sequencing.

### Total bacterial DNA (qPCR) and detection of Mollicutes (PCR)

#### Quantitative PCR (qPCR)

Total bacterial DNA quantity in each sample was estimated using a SYBR Green assay based on amplification of the V3 region of the 16S rRNA gene. Primer sequences were as follows: SRV3-1 (5′-CGGYCCAGACTCCTAC-3′), SRV3-2 (5′-TTACCGCGGCTGCTGGCAC-3′) [36]. Reactions were run on a MyiQ thermocycler using the following cycling parameters: 95 °C for 3 min, followed by 30 cycles of 95 °C for 15 s, 62 °C for 15 s and 72 °C for 15 s, with a final extension at 72 °C for 5 min [37].

#### Conventional PCR

Some Mollicutes (*Mycoplasma* and *Ureaplasma*) species lack a *cpn*60 gene [38]. Thus, we performed a family-specific semi-nested PCR targeting the 16S rRNA gene to detect Mollicutes [39], and a PCR targeting the multiple-banded antigen gene to detect *Ureaplasma* spp. PCR products from *U. parvum* and *U. urealyticum* can be differentiated by size [40].

#### *cpn*60 universal target (UT) PCR and pyrosequencing

Universal primer PCR targeting the 549–567 bp *cpn*60 UT region was performed using a mixture of *cpn*60 primers consisting of a 1:3 M ratio of primers H279/H280:H1612/H1613, as described previously [41–43]. To allow multiplexing of samples in a single sequencing run, primers were modified at the 5′ end with one of 24 unique decamer multiplexing identification (MID) sequences, as per the manufacturer’s recommendations (Roche, Brandford, CT, USA). Amplicons were pooled in equimolar amounts for sequencing on the Roche GS Junior sequencing platform. The sequencing libraries were prepared using the GS DNA library preparation kit, and emulsion PCR (emPCR) was performed with a GS emPCR kit (Roche Diagnostics, Laval, Canada).

Samples were handled in small batches to avoid cross-contamination, and experimental controls were included at several steps in the study. Regular monitoring of DNA extraction controls in our lab by universal PCR confirms that these procedures are sufficient to eliminate detectable template contamination of study samples. A no template control was also included in each set of PCR reaction as negative controls. Experimental controls were not sequenced as they did not yield any amplification.

#### Analysis of operational taxonomic units (OTU)

Raw sequence data was processed by using the default on-rig procedures from 454/Roche. Filter-passing reads were used in the subsequent analyses for each of the pyrosequencing libraries. MID-partitioned sequences were mapped using Bowtie 2 (http://bowtie-bio.sourceforge.net/bowtie2/) on to a manually curated reference set of 1561 OTU sequences representing the human vaginal microbiota. Bowtie 2 was run using the default end-to-end alignment mode.

The OTU reference set was generated originally by de novo assembly of *cpn*60 sequence reads from 546 vaginal microbiomes using the microbial Profiling Using Metagenomic Assembly pipeline (mPUMA, http://mpuma.sourceforge.net) [44] with Trinity as the assembly tool [45] (Additional file 1). OTU were labeled according to their nearest reference sequence determined by watered-Blast comparison [46] to the *cpn*60 reference database, cpnDB_nr (downloaded from http://www.cpndb.ca [38]). This reference assembly approach allows us to compare the microbial profiles from various cohorts under investigation, including the 46 pregnant women who had sPTB described in this study.

The result of mapping is an OTU frequency table (Additional file 2) that was used for microbiome data analysis. Some analyses were also performed at species level, i.e. combined OTU that have the same nearest neighbour.

#### Statistical analysis

Comparisons of socio-demographic characteristics of cohorts and participants were based on analysis of variance (ANOVA), *t* test and chi-square, performed in IBM SPSS (Statistical Package for the Social Sciences, version 21) at 5% level of significance. For analysis of associations between socio-demographic characteristics and microbiota profiles (CST), a false discovery rate (FDR) correction for multiple comparisons was applied [47].

Alpha (Shannon diversity and Chao1 estimated species richness) and beta diversity (jackknifed Bray–Curtis dissimilarity matrices) were calculated as the mean of 100 subsamplings of 1000 reads (or all reads available when less than 1000) in QIIME (Quantitative Insights Into Microbial Ecology) [48]. Plots of alpha diversity measures against bootstrap sample number were generated in R and visually inspected to ensure that an adequate sampling depth for each sample was achieved.

For community state type (CST) analysis, a Jensen–Shannon distance matrix was calculated using the ‘vegdist’ function in the *vegan* package [49] with a custom distance function that calculates the square root of the Jensen–Shannon divergence [50]. This distance matrix was used for hierarchical clustering using the ‘hclust’ function in R, with Ward linkage.

The function *aldex.clr* from the ALDEx2 package in R was used to compare the differential relative abundance of individual taxa in term and preterm groups [51]. Significant differences were determined based on the false discovery rate (FDR), which is the result of a Benjamini–Hochberg corrected *p* value from a Welch’s *t* test calculated within ALDEx2.

## Results

### Description of the study population and pregnancy outcomes

Socio-demographic characteristics of women who had spontaneous preterm birth (*n* = 46) and women who had term deliveries (*n* = 170) are summarized in Table 1. There were no significant differences in maternal age, BMI, ethnicity, smoking status, consumption of alcohol or use of probiotics between term and preterm groups (all *p* > 0.05). Average maternal age was 33 for participants in both cohorts. Average body mass index (BMI) was 22.9 and 24.2 for women in the term and preterm groups, respectively. Most women in both cohorts identified themselves as white ethnicity, followed by East Asian and South/Southeast Asian (Table 1). Consumption of tobacco (term 2.3%; preterm 0%), alcohol (term 5.9%; preterm 4.3%) or probiotic supplements (term 4.1%; preterm 6.5%) was low among women in both groups (chi-square, all *p* > 0.05).

**Table 1:** Socio-demographic and microbiological characteristics of subjects

Characteristics	Term pregnancies (*n* = 170)	Preterm pregnancies (*n* = 46)	*p* value
Age (mean ± SD, range)^1^	33.6 ± 4.2 (21–45)	33.65 ± 4.1 (25–45)	0.948
21–25	5 (2.9%)	1 (2.1%)	
26–35	114 (67.1%)	32 (69.5%)	
36–45	51 (30.0%)	13 (28.2%)	
BMI (mean ± SD, range)^1^	22.9 ± 3.8 (17–40)	24.2 ± 5.6 (19–43)	0.125
Underweight (< 18.50)	7 (4.1%)	0 (0%)	
Normal weight (18.51–24.9)	131 (77.0%)	33 (73.3%)	
Overweight (25.0–29.9)	25 (14.7%)	8 (17.7%)	
Obese (> 30)	7 (4.1%)	4 (9.0%)	
MD^3^	0 (0%)	1 (2.2%)	
Ethnicity^2^			0.261
White	108 (63.5%)	22 (47.8%)	
East Asian	26 (15.3%)	6 (13.0%)	
South/Southeast Asian	15 (8.8%)	4 (8.7%)	
Latin America/Hispanic	8 (4.7%)	3 (6.5%)	
Black	3 (1.8%)	2 (4.4%)	
Other/mixed ethnicity	10 (5.9%)	6 (13.0%)	
MD	0 (0%)	3 (6.5%)	
Community state type (CST)^2^			0.361
I	56 (32.9%)	17 (37%)	
II	9 (5.3%)	5 (10.9%)	
III	28 (16.5%)	8 (17.4%)	
IVA	31 (18.2%)	6 (13%)	
IVC	19 (11.2%)	2 (4.3%)	
IVD	11 (6.5%)	1 (2.2%)	
V	16 (9.4%)	7 (15.2%)	
Estimated bacterial load (log copies of 16S rRNA gene)/swab (mean ± SD, range)^1^	7.78 ± 0.93 (4.89–10.67)	8.07 ± 0.71 (6.32–10.33)	0.049
Presence of Mollicutes^2^	68 (40%)	28 (60.8%)	0.012
Presence of *Ureaplasma*^2^	40 (23.4%)	14 (30.4%)	0.337
*U. parvum*	37 (21.7%)	14 (30.4%)	
*U. urealyticum*	3 (1.7%)	0 (0%)	
Shannon diversity (mean ± SD, range)^1^	1.28 ± 0.86 (0.13–4.52)	1.81 ± 1.13 (0.34–5.16)	0.004
Chao1 richness (mean ± SD, range)^1^	36.22 ± 14.80 (14.39–115.74)	46.38 ± 24.19 (20.20–126.01)	0.009

^1^*t* test; ^2^chi-square; ^3^MD = missing data

Most women in the preterm group had a Bachelor/graduate degree (29/46) and an average house income higher than CAD 100,000 per year (25/46). A minority of women who had preterm birth (5/46) reported consumption of substances without prescription prior pregnancy, of which 3/46 women consumed marijuana/hashish, 1/46 woman consumed tranquilizers/nerve pills and 1/46 woman consumed cocaine/crack. Approximately 74% of the participants in the preterm group reported a pre-existing condition. A total of 12/46 women had some condition related to mental health, such depression or anxiety. Seventeen percent (8/46) had a neurological condition, including migraine headaches, and 24% (11/46) had a genitourinary condition (corpus luteal cyst, bicornuate uterus, cervical polyp, cervical dysplasia (2), uterine polyp, ovarian cyst, polycystic ovarian syndrome, urinary tract infections with and without kidney stones (3).

Characteristics regarding pregnancy and neonatal outcomes are described in Table 2. Pregnancy outcome information was not available for one woman in the preterm group as she was lost to follow-up. There were no significant differences in gestational age at enrolment, mode of conception or fetal sex between groups (all *p* > 0.05). Average gestational age at delivery was 39^+3^ weeks for the women who delivered at term and 34^+2^ weeks for women who had preterm birth, most of which were considered late preterm, i.e. delivery between 32 and 36^+6^ weeks of gestational age. Women in the preterm group were more likely to have experienced preterm birth or miscarriage in their previous pregnancy (chi-square, *p* < 0.001). They also had higher percentage of caesarean sections than women who delivered at term. Number of previous gestations also differed between groups; women who had preterm birth were more likely to be primigravida (22/46) in comparison with women who had term deliveries (45/170). There was a significant difference between term and preterm groups regarding birth weight and number of infants admitted to level 3 neonatal intensive care unit (NICU) (Table 2). Apgar score at 1 (term 8.75 ± 0.6; preterm 8.38 ± 1.1) and 5 min (term 8.97 ± 0.17; preterm 8.76 ± 0.7) between groups also differed (*t* test, all *p* < 0.001). One preterm infant (1/46) died shortly after birth (20 weeks of gestational age).

**Table 2:** Gestation characteristics, pregnancy and neonatal outcomes

Characteristics	Term pregnancies (*n* = 170)	Preterm pregnancies (*n* = 46)	*p* value
Gestational age in weeks^+day^			
At enrolment (mean ± SD, range)^1^	13^+ 2^ ± 1^+1^ (11^+1^–16^+6^)	13^+3^ ± 1^+0^ (11^+6^–16^+0^)	0.641
At delivery (mean ± SD, range) ^1^	39^+3^ ± 0^+6^ (39^+3^–41^+2^)	34^+2^ ± 2^+6^ (20^+0^–36^+6^)	< 0.0001
Late preterm (32 to 36^+6^)	NA	39 (84.8%)	
Very preterm (28 to 31^+6^)	NA	5 (10.8%)	
Extremely early (≤ 27^+6^)	NA	1 (2.1%)	
Previous pregnancy history (excludes women in first pregnancy)^2^	(*n* = 125)	(*n* = 24)	< 0.0001
Livebirth, term	89 (71.2%)	9 (37.5%)	
Livebirth, preterm	0 (0%)	3 (12.5%)	< 0.0001
Spontaneous abortion	20 (16%)	8 (33.3%)	0.046
Pregnancy termination	16 (12.8%)	3 (12.5%)	0.968
Ectopic pregnancy	0 (0%)	1 (4.1%)	
Mode of delivery^2^			0.042
Vaginal delivery	128 (75.3%)	27 (58.7%)	
C-section	42 (24.7%)	18 (39.1%)	
Parity^2^			0.005
0	55 (32.3%)	28 (60.8%)	
1	94 (55.3%)	12 (26%)	
2–4	21 (12.3%)	6 (13%)	
Assisted conception^2^	17 (10%)	6 (13%)	0.591
Fetal sex (% male/% female) ^2^	(48.8%)/(51.1%)	(41.3%)/(56.5%)	0.430
Birth weight (g) (mean ± SD, range)^1^	3398 ± 459 (1970–5200)	2550 ± 559 (1300–3595)	< 0.0001
Infant in NICU (*n*, %)^2^	1 (0.6%)	24 (52.2%)	< 0.0001

^1^*t* test; ^2^chi-square

Among women who delivered preterm, 63% (29/46) had premature rupture of membranes (PPROM), 10.8% (5/46) had gestational diabetes and 4.3% (2/46) had anemia unresponsive to therapy. Twenty-four percent of women (11/46) presented one of the following conditions: maternal elevated liver enzymes, short cervix and incompetent cervix; fetal ascites, fetal distress and large foetus for gestational age; and placental findings of marginal cord insertion, two-vessel umbilical cord, placenta previa and low-lying placenta.

### Sequencing results and OTU analysis

Raw sequence data files for the samples described in this study were deposited to the NCBI Sequence Read Archive (Accession SRP073152, BioProject PRJNA317763; BioProject PRJNA403856). Total dataset contained 1,635,072 *cpn*60 reads; median and average read count per sample were 4936 and 7569 (range 402–37,378), respectively. Average read length was 424 bp. Results of Bowtie2 mapping showed that these reads corresponded to 728 OTUs from the reference assembly (Additional file 1).

### Microbiota profiles

**Fig. 1:**
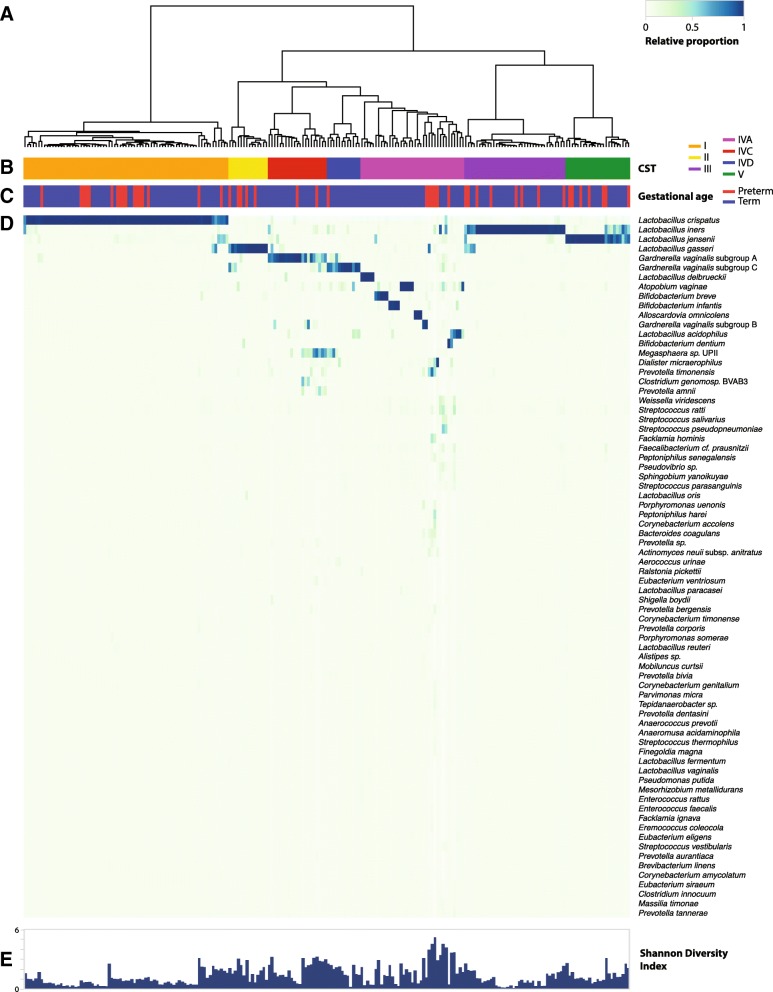
Vaginal microbiota profiles of women who had sPTB and term deliveries. **a** Hierarchical clustering of Jensen–Shannon distance matrices with Ward linkage on the relative proportions of reads for each OTU within individual vaginal samples. **b** Community state type (CST). **c** Gestational age at delivery. **d** Heatmap of relative abundances of bacterial species within each vaginal microbiota. Each column represents a woman’s vaginal microbiota profile, and each row represents a bacteria species. Only species that are at least 1% abundant in at least one sample are shown. **e** Shannon diversity indices calculated for each sample

Microbiota profiles were created by PCR amplification and pyrosequencing of the *cpn*60 universal target region. Hierarchical clustering of vaginal microbiota profiles resulted in seven community state types (CST): I (*Lactobacillus crispatus* dominated), II (*Lactobacillus gasseri* dominated), III (*Lactobacillus iners* dominated), IVA (*Gardnerella vaginalis* subgroup B or mix of different species), IVC (*G. vaginalis* subgroup A dominated), IVD (*G. vaginalis* subgroup C dominated) and V (*Lactobacillus jensenii* dominated) (Fig. 1). Each CST is defined by the dominance of one species of *Lactobacillus* (I, II, III, V), *Gardnerella vaginalis* (IVC, IVD) or a mixture of bacteria species (IVA), as previously described [52, 53].

**Fig. 2:**
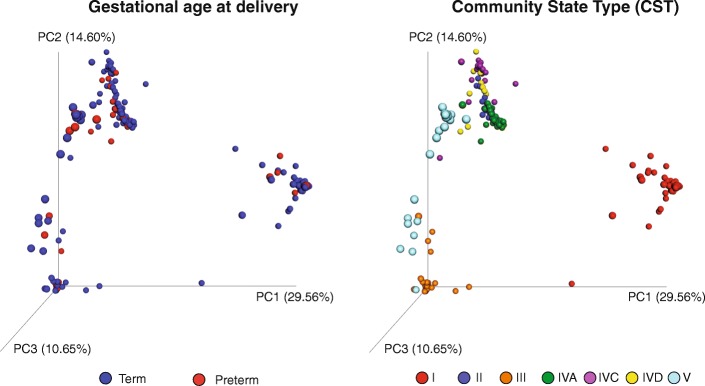
Vaginal microbiota profiles coloured by gestational age at delivery or CST. Jackknifed principal coordinates analysis (PCoA) of Bray–Curtis distance matrices of microbial profiles from all participants in the study

Overall microbiota profiles did not cluster together based on gestational age at delivery (Figs. 1 and 2). Most microbial profiles from the preterm group (80.5%) were assigned to *Latobacillus*-dominated CST: CST I (37% of profiles), CST III (17.4%), CST V (15.2%) and CST II (10.9%). The remaining profiles (19.5%) were assigned to CST IVA, IVC or IVD (Table 1). The CST IVA was the most heterogeneous group, represented by the dominance of *Lactobacillus delbrueckii*, *Bifidobacterium dentium*, *Bifidobacterium infantis*, *Atopobium vaginae*, *Bifidobacterium breve* or a mixture of different bacteria species. The CST IVC was dominated by *G. vaginalis* subgroup A and *Megasphaera* spp., and CST IVD was dominated by *G. vaginalis* subgroup C (Fig. 1).

### Ecological analysis and total bacterial load

Assessment of alpha diversity revealed that microbiomes of women who delivered preterm were richer (Chao1 richness 46.3 ± 24.1) and more diverse (Shannon diversity index 1.8 ± 1.1) when compared to those of women in the term group (36.2 ± 14.8; 1.2 ± 0.8) (*t* test, *p* < 0.01) (Table 1). Total bacterial load was estimated based on qPCR targeting the 16S rRNA gene, and it was expressed as log 16S rRNA gene copy number per swab. Higher bacterial loads were detected in samples from the preterm group (7.7 ± 0.9) compared to term group (8.0 ± 0.7) (*t* test, *p* = 0.049) (Table 1).

### Bacteria species relative abundance and prevalence

**Fig. 3:**
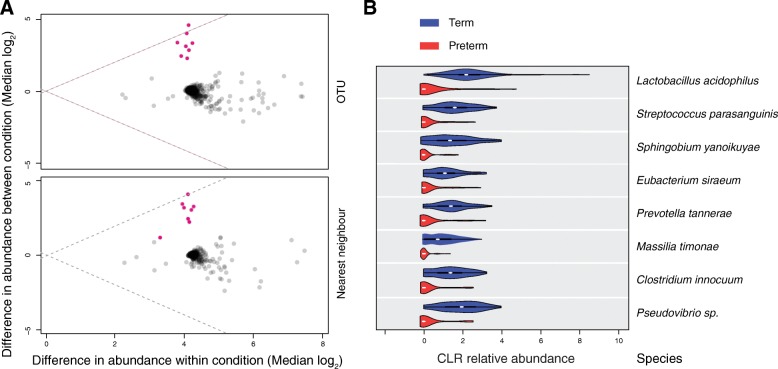
Bacteria relative abundance differences between term and preterm groups represented by ALDEx2. **a** ALDEx2 between- and within-difference values for individual organisms across gestational age category. Organisms (at OTU and nearest neighbour species level) with significant *p* values are shown as pink circles (Welch’s *t* statistical test). **b** Violin plots showing the bacteria relative abundance (centre log transformed, CLR) in term and preterm groups. Only the eight bacteria with significant relative abundance differences between term and preterm groups are shown. In the violin plots, the white dot represents the median value, the black bar is the interquartile range, and the vertical width of the plot shows the density of the data along the *X*-axis

To investigate whether there was an association between individual taxa and sPTB, the abundance and prevalence of each species was evaluated. The ALDEx2 analysis assessed the relative abundance of each taxa (at the OTU and species level) in term and preterm groups. Eight OTU/species were more abundant in the term group in comparison with preterm, all of which were considered rare members of the bacterial community (Fig. 3). *L. acidophilus* represented 1% of the total reads in the dataset and had a low relative abundance average of 1.98% (range 0–69%) and 0.18% (range 0–0.87%) in samples from term and preterm groups respectively. All the other seven bacteria together represented only 0.4% of the total reads in the dataset.

Bacteria prevalence (presence/absence) was also assessed (only species with at least 10 total reads were included). A total of 60 taxa had significant differences in prevalence between term and preterm groups; 11 species had greater prevalence in the term cohort and 49 species were more prevalent in the preterm cohort (Table 3). *Bifidobacterium infantis*, for example, was two times more prevalent in the term group in comparison with preterm, and *Prevotella timonensis* was 1.58 times more prevalent in the preterm group (Table 3). Several *Prevotella* spp. were associated with both term and preterm. *Prevotella amnii* and *P. tannerae* had greater prevalence in the term cohort, whereas *P. timonensis*, *P. bivia*, *P. corporis* and *P. bucalis* were more prevalent in the preterm group (Table 3). It is important to note that read depth distribution did not differ between term and preterm cohorts (*t* test, *p* > 0.05); therefore, the differences observed here in bacteria prevalence were unlikely to be driven by cohort sequencing bias.

**Table 3:** Bacteria prevalence in the vaginal microbiomes of women who delivered preterm and at term

Species	Total reads	Preterm (*n* = 46)		Term (*n* = 170)		Prevalence ratio	FDR*
		Reads	Prevalence (%)	Reads	Prevalence (%)		
A						term/preterm	
*Bifidobacterium infantis*	56,246	409	19.57	55,837	40.00	2.04	0.033
*Lactobacillus delbrueckii* subsp*. lactis*	48,063	9	4.35	48,054	28.82	6.63	0.005
*Lactobacillus acidophilus*	17,440	569	89.13	16,871	97.65	1.10	0.033
*Prevotella amnii*	13,339	68	4.35	13,271	21.76	5.01	0.023
*Pseudovibrio* sp.	1634	52	15.22	1582	91.76	6.03	0.000
*Streptococcus parasanguinis*	1199	63	19.57	1136	88.82	4.54	0.000
*Sphingobium yanoikuyae*	1174	16	13.04	1158	80.00	6.13	0.000
*Prevotella tannerae*	930	58	15.22	872	88.24	5.80	0.000
*Clostridium innocuum*	814	42	13.04	772	81.18	6.22	0.000
*Eubacterium siraeum*	641	43	17.39	598	78.24	4.50	0.000
*Massilia timonae*	426	6	8.70	420	70.00	8.05	0.000
B						preterm/term	
*Prevotella timonensis*	11,450	4211	71.74	7239	45.29	1.58	0.005
*Dialister micraerophilus*	7381	2850	67.39	4531	44.12	1.53	0.021
*Prevotella* sp.	2216	626	47.83	1590	14.71	3.25	0.000
*Bacteroides coagulans*	1283	1212	41.30	71	7.06	5.85	0.000
*Corynebacterium accolens*	1083	999	52.17	84	13.53	3.86	0.000
*Porphyromonas uenonis*	1040	795	28.26	245	12.94	2.18	0.038
*Actinomyces neuii* subsp*. anitratus*	877	738	43.48	139	10.00	4.35	0.000
*Peptoniphilus harei*	875	811	45.65	64	7.65	5.97	0.000
*Lactobacillus fermentum*	729	664	17.39	65	5.29	3.29	0.026
*Facklamia hominis*	711	709	15.22	2	1.18	12.93	0.000
*Prevotella bivia*	638	228	26.09	410	8.24	3.17	0.005
*Prevotella corporis*	535	463	23.91	72	5.88	4.07	0.000
*Corynebacterium timonense*	503	355	34.78	148	10.00	3.48	0.000
*Corynebacterium genitalium*	372	323	34.78	49	7.06	4.93	0.000
*Tepidanaerobacter* sp.	329	303	23.91	26	4.12	5.81	0.000
*Corynebacterium amycolatum*	264	228	32.61	36	4.71	6.93	0.000
*Parvimonas micra*	193	183	10.87	10	1.18	9.24	0.005
*Mobiluncus curtsii* subsp. *curtsii*	183	171	21.74	12	2.35	9.24	0.000
*Finegoldia magna*	155	149	13.04	6	3.53	3.70	0.038
*Coprococcus eutactus*	154	68	47.83	86	27.06	1.77	0.026
*Brevibacterium linens*	147	142	10.87	5	0.59	18.48	0.000
*Rothia dentocariosa*	144	120	30.43	24	5.29	5.75	0.000
*Streptococcus thermophilus*	132	126	19.57	6	2.35	8.32	0.000
*Magnetospirillum magnetotacticum*	116	40	43.48	76	24.12	1.80	0.033
*Dethiobacter alkaliphilus*	101	97	10.87	4	1.76	6.16	0.017
*Brevibacterium massiliense*	99	86	15.22	13	1.18	12.93	0.000
*Eremococcus coleocola*	90	82	10.87	8	1.76	6.16	0.017
*Anaeromusa acidaminophila*	86	85	15.22	1	0.59	25.87	0.000
*Arthrobacter globiformis*	66	48	10.87	18	1.76	6.16	0.017
*Staphylococcus epidermidis*	54	47	13.04	7	2.35	5.54	0.009
*Corynebacterium simulans*	52	41	26.09	11	1.76	14.78	0.000
*Anaeroglobus geminatus*	52	49	15.22	3	1.18	12.93	0.000
*Cellvibrio gilvus*	42	26	15.22	16	1.76	8.62	0.000
*Prosthecochloris vibrioformis*	41	40	6.52	1	0.59	11.09	0.028
*Prevotella buccalis*	33	33	15.22	0	0.00	–	0.000
*Acidaminococcus fermentans*	31	29	13.04	2	1.18	11.09	0.000
*Brevibacterium casei*	25	24	8.70	1	0.59	14.78	0.005
*Streptococcus sanguinis*	23	18	13.04	5	2.35	5.54	0.009
*Atopobium parvulum*	23	20	8.70	3	1.18	7.39	0.023
*Peptoniphilus duerdenii*	22	20	10.87	2	0.59	18.48	0.000
*Pelobacter propionicus*	21	18	6.52	3	0.59	11.09	0.028
*Staphylococcus hominis*	21	17	15.22	4	1.76	8.62	0.000
*Atopostipes suicloacalis*	18	8	10.87	10	1.18	9.24	0.005
*Corynebacterium pyruviciproducens*	18	13	13.04	5	1.76	7.39	0.005
*Corynebacterium jeikeium*	14	11	13.04	3	0.59	22.17	0.000
*Sporichthya polymorpha*	13	11	8.70	2	1.18	7.39	0.023
*Rhodococcus jostii*	13	7	8.70	6	1.18	7.39	0.023
*Nitrospina gracilis*	10	5	8.70	5	0.59	14.78	0.005
*Megasphaera* sp. BV3C16-1	10	9	6.52	1	0.59	11.09	0.028

Panel A: species with greater prevalence in the term group (ratio term/preterm)

Panel B: species with greater prevalence in the preterm group (ratio preterm/term)

*FDR (false discovery rate) represents the corrected *p* value for multiple comparisons

Mollicutes (*Mycoplasma* and/or *Ureaplasma*) were detected by family-specific conventional PCR in 28/46 (60%) of pregnant women who delivered preterm (Table 1). *Ureaplasma* species were detected by genus-specific PCR in samples of 14/46 (30%) women who had PTB, with all women testing positive for *U. parvum* and none for *U. urealyticum*. Women who delivered at term were less likely to be PCR positive for Mollicutes compared to women who had PTB (Table 1). No significant differences were observed in *Ureaplasma* prevalence between the two groups (Table 1). An association between Mollicutes/*Ureaplasma* detection and the composition of the vaginal microbiota, represented as CST, was also investigated. Detection of Mollicutes and *Ureaplasma* was not associated with any CST in particular when investigated in the term cohort, preterm cohort or both groups together (chi-square, *p* > 0.05).

### Relationships between microbiological and socio-demographic characteristics within the preterm group

The association between CST (I, II, III, IVC, IVD, V) from profiles of women who delivered preterm and several microbiologic-socio-demographic characteristics was investigated. Only two associations were significant: CST and microbiota richness, and CST and microbiota diversity (ANOVA, *p* < 0.001). There was no significant association between CST and the remaining following metadata: microbiota richness and diversity (continuous variable), presence of Mollicutes and *Ureaplasma* (yes/no), log 16S rRNA gene copies (continuous variable), maternal age (continuous variable; 18–25, 26–35, 36–45), BMI category (underweight, normal, overweight, obese; < 25, ≥ 25), ethnicity (White, East Asian, South Asian, Black, Hispanic, Other), natural conception (yes/no), parity (0, > 1), gestational age (continuous variable), mode of delivery (vaginal, C-section), pre-existing condition (yes/no), folic acid intake before or during pregnancy (yes/no), drinking alcohol (yes/no), neonate in high level care (yes/no), birth weight (continuous variable) and Apgar score at 1 and 5 min (1–9).

## Discussion

In this study, we determined the composition of the vaginal microbiota of women who had spontaneous preterm birth and compared these profiles to those of women who delivered at term, previously reported by our research group [24]. The availability of foundational data on women who delivered at term and the infeasibility of collecting large numbers of samples at 11–16 weeks gestation from women who would go on to deliver pre-term, our study design included comparison of samples collected in a previously published study [24] and from the OBS. To minimize any batch effects, we were rigorous in implementation of consistent sample processing and did extensive analysis of the clinical and demographic characteristics to ensure they were well matched (Table 1). The cohorts were comparable in terms of maternal age, BMI, ethnicity, consumption of tobacco, alcohol and probiotics, which is of interest given that several of these characteristics have been previously associated with preterm delivery. In particular, previous described factors included low and high maternal ages [5–7], low BMI [8], black ethnicity [9], high levels of tobacco, alcohol and illicit drugs consumption [4], close temporal proximity to a previous delivery [10] and multiple gestation [11]. This cohort is unique in that it did offer the opportunity to have gestational age at delivery as the main characteristic distinguishing these two groups recognizing that the majority of preterm births occurred beyond 32 weeks gestation.

A difference in number of previous gestations was observed between groups, with women who experienced preterm birth more likely to be primigravida in comparison with women who had term deliveries. It has been recently demonstrated that women with a prior conception, regardless of whether or not this proceeded to a birth, have a decrease in the relative abundance of *L. crispatus* and a concomitant increase in the abundance of other *Lactobacillus* species as well as *Gardnerella* [54].

Other known risk factors for sPTB include maternal medical disorders like hypertension, asthma, diabetes and thyroid disease [4]. Although some women in both cohorts reported these conditions, there were not enough participants to stratify the data based on the individual disorder and therefore was not possible to investigate the interaction between those medical conditions and gestation outcome. We were, however, able to confirm previous reports of history of prematurity as a risk factor for preterm birth [55].

Since many organisms isolated from the amniotic cavity of women who experienced preterm birth are also found in the genital tract [12–15], an intrauterine infection ascending from the vagina is one of the currently hypothesized triggers of PTB [56]. In this study, however, we did not identify a signature microbiota composition (CST) associated with preterm birth. This observation is consistent with the results presented by others [29, 30]. CST assignments are largely driven by the dominance of a single species, which may mask differences in rare taxa that would differentiate term and preterm groups, and indeed, further analysis revealed that the vaginal microbiota of women who experienced preterm birth was richer and more diverse than those of women who delivered at term. Also, most women (84.8%) in our study were considered late preterm and although we cannot address this question, it is possible that sPTB driven by an ascending infection would be more evident in a high-risk cohort or extreme preterm cases. A recent study of a high-risk pregnant cohort has reported that *L. iners* was strongly associated with short cervix and preterm birth, as *L. crispatus* was associated with term deliveries [57]. Those differences in study outcomes indicate that the pathogenesis of sPTB in low- and high-risk groups might be different. Identifying differences in the causes of early and late sPTB and the role of the vaginal microbiota in those processes will require further study.

One controversy that challenges the current hypothesis of preterm caused by an ascending infection is that antibiotic administration to pregnant women with a disturbed vaginal microbiota does not improve outcome in most cases, as demonstrated by study trials [58, 59] and systematic reviews [60–62]. One explanation for the inefficacy of antibiotic treatment in the prevention of preterm birth relies is the high rates of antibiotic resistance among bacterial-vaginosis-associated bacteria [63, 64]. In this case, antibiotics not only do not kill the targeted bacteria, but might also reduce the vaginal *Lactobacillus* population leading to an even more disturbed microbiota, as recently demonstrated [65].

In addition to differences in richness and diversity, differences in the microbiota between the two cohorts regarding bacterial abundance and prevalence were also identified. The ALDEx2 analysis indicated that eight rare taxa were more abundant in the term group, which does not necessarily mean they are associated with a ‘healthier’ state or implicated in preventing sPTB. Since these bacteria are detected at very low abundance within the microbiota profiles, their biological significance in the vaginal microbiome is questionable. Differences in the prevalence of several other taxa between groups were also observed. For example, more women in the term group had *Prevotella amnii* and *P. tannerae* detected in their vaginal samples, whereas *P. timonensis*, *P. bivia*, *P. corporis* and *P. bucalis* were more frequently detected in samples from women in the preterm group (Table 3). *Prevotella* spp. have been previously associated with bacterial vaginosis and preterm labour [22, 66, 67], and our results indicate that different *Prevotella* species might have different roles in sPTB. Several of the taxa that were significantly different in their prevalence among women in the two groups also had low sequence read counts (Table 3). Further investigation would be required to determine if these rare members of the microbial community play a yet unknown role in sPTB.

It is also important to note that the number of bacterial species with greater prevalence in the preterm (49/60) was higher than in the term (11/60) cohort (Table 3), which is consistent with our results of increased microbial richness and diversity in the samples from women who experienced preterm birth. This might indicate that increased richness, rather than the presence of specific taxa, might be associated with sPTB. Those differences might also be an indicator of physiological/biochemical dissimilarities in the vaginal microbiomes of women who deliver at term or preterm. In other words, the physiological state that leads to sPTB might also create an environment that supports a richer/more diverse microbiota.

Our results also confirmed previous reports of an association between *Mycoplasma* and preterm birth [68]. Mollicutes were detected significantly more often in women in the preterm group compared to women in the term group, but no differences were observed in *Ureaplasma* prevalence between groups indicating that the difference in Mollicutes prevalence is primarily driven by the presence of *Mycoplasma* spp. Although individual *Mycoplasma* species could not be discerned based on assay used in our study, both *Mycoplasma genitalium* [69–71] and *Mycoplasma hominis* [72–75] have been previously associated with negative reproductive outcomes including PTB.

Collectively, our overall findings were similar to other two studies, which provided us the opportunity to compare different study designs (based on different cohorts and barcode gene) that addressed the same research question. Hyman and colleagues [30] described the vaginal microbiota of 83 pregnant women (term *n* = 66, preterm *n* = 17) based on Sanger sequencing of cloned 16S rRNA genes. Samples were collected at each trimester and preterm was defined as delivery before 37 weeks of gestation. There was no correlation between preterm and absence/low abundance of *Lactobacillus* in the microbiota; in other words, preterm outcome could not be predicted based on CST. Similar to our results, they found an association between increased microbiota diversity and preterm delivery among women of white ethnicity (*n* = 40) (data from women of others ethnicities was not included in the analysis because of small sample sizes). Although there was no association between CST and ethnicity, it is important to note that most women enrolled in this study described themselves as being white, and it is possible that an increased sample size of participants of other ethnicities could result in a different conclusion.

Romero and colleagues [29] also investigated the vaginal microbiota of pregnant women who experienced preterm, defined as delivery before 34 weeks of gestation (term *n* = 72, preterm *n* = 18). The profiles were created by 16S rRNA amplicon sequencing, and samples were collected every 4 weeks until 24 weeks of gestation and then every 2 weeks. They found no differences in the frequency of different CST between women who had term and preterm deliveries. Likewise, no differences in bacteria relative abundance were observed between the two cohorts, although only bacteria that were present in at least 25% of samples were included in the analysis. These results are consistent with our findings of bacterial abundance based on the ALDEx analysis since we only found significant differences in relative abundance for eight rare bacteria. Unlike Hyman et al. [30] and our results, Romero et al. [29] did not find differences in microbiota diversity between women who delivered preterm and at term. One possible explanation for this contradictory result might be related to differences in participant ethnicity among these studies. While most women in our study and the Hyman et al. study described themselves as white, the majority of participants in the Romero et al. study described themselves as African American. It has been reported that the composition of the vaginal microbiota is strongly associated with a woman’s ethnicity [52, 76]. Other studies have also demonstrated that black ethnicity is associated with an increased microbiota diversity in comparison with white ethnicity [77], which could have masked differences in bacterial diversity between term and preterm cohorts in the Romero study.

Contrary to our overall findings, DiGiulio and colleagues [31] found a strong association between the non-*Lactobacillus*-dominated CST IV and preterm birth in a case–control study based on the 16S rRNA amplicon sequencing. Pregnant women (preterm *n* = 34, term *n* = 15), mostly of white ethnicity, were sampled weekly throughout gestation. Interestingly, the authors pointed out that if samples had been collected less frequently, short-term ‘excursions’ to CST IV would have been missed and probably the association between CST IV and preterm birth would have been less obvious. The detection of a temporary microbiota disturbance represented by a change from a *Lactobacillus*-dominated CST to CST IV may have been missed in our study since samples were not collected longitudinally. Moreover, a recent study has demonstrated that PTB–microbiota associations are population-dependent [32]; lower *Lactobacillus* and higher *Gardnerella* abundances were associated with PTB in a low-risk predominantly Caucasian cohort, but not in a high-risk predominantly African American cohort. These population-dependent associations might contribute to explain contradictory conclusions among different studies and emphasize the importance of investigating the vaginal microbiota of different populations with varying ethnic backgrounds and from different geographical locations.

Most samples in the preterm group were dominated by *Lactobacillus*, yet, they collectively had higher richness and diversity compared to samples from the term group. The increased microbiota richness/diversity might indicate a transient state between *Lactobacillus*-dominated CST and non-*Lactobacillus*-dominated, i.e., CST IV (A, C or D). In other words, the increased richness and diversity we observed might be a remnant characteristic of the previous disturbed microbiota. In summary, although we did not “detect” a specific microbial community structure that is associated with preterm birth, the increased microbiota richness/diversity was associated with preterm birth. In addition, the association with differences in *Prevotella* species and *Mycoplasma* presence may point to signature species associated with preterm birth.

## Conclusions

Taken together, our results suggest that the differences in the microbiota of women who had preterm deliveries, such as increased microbiota richness and diversity and greater prevalence of Mollicutes and other bacteria, may have a role in sPTB. Other differences between cohorts might have been masked by the presence of highly dominant bacteria like *Lactobacillus*. At the overall level, we did not identify a specific vaginal microbial community structure at 11–16 weeks gestation age that predicts sPTB. Also, differences in relative abundance of bacterial species between term and preterm groups were only significant for a few low abundance species. Although a causal relationship remains to be determined, our results confirm previous reports of an association between Mollicutes and preterm birth, and further suggest that a diverse bacterial community may contribute to the microbiome’s role in sPTB. Alternatively, the more rich and diverse microbiotas of the preterm group may reflect physiological differences between the groups that affect selection of bacteria. This study provides valuable evidence of subtle alterations in the microbiome associated with preterm birth that requires further study utilizing sequencing methodology. In addition, future study should include evaluation of the microbial metabolite production and host response to further elucidate factors leading to sPTB and identify women at risk early in pregnancy.

## Additional files

Additional file 1: *cpn*60 OTU sequences. Multiple fasta file containing 728 OTU sequences. (TXT 336 kb)

Additional file 2: Summary of OTU analysed in this study. OTU ID, percentage of identity, length, cpnDB name, species, and abundance in each library are shown. (XLSX 500 kb)
